# Hibernation Shifts in Gut Microbiota Composition and Metabolic Function in the Chinese Horseshoe Bat (*Rhinolophus sinicus*)

**DOI:** 10.1002/ece3.73087

**Published:** 2026-02-10

**Authors:** Weiwei Shao, Yalei Li, Xiaoyun Cheng, Ling Guo, Li Wei

**Affiliations:** ^1^ College of Agriculture and Biotechnology Lishui University Lishui China; ^2^ Suichang Forestry Development Center Suichang China

**Keywords:** 16S rRNA, Chiroptera, gut microbiota, *Rhinolophus sinicus*, seasonal changes

## Abstract

The composition and function of animal gut microbiota are influenced by various intrinsic and extrinsic factors. Hibernation represents a significant physiological challenge for heterothermic mammals, yet the effects on gut microbiota in bats remain understudied. This study investigated seasonal variations in the gut microbiota of *Rhinolophus sinicus* between summer activity and winter hibernation using 16S rRNA gene sequencing (*n* = 12 per group). Sequencing analysis identified 907 ASVs in the hibernation group and 555 ASVs in the summer group, with only 27 ASVs shared between groups, suggesting substantial seasonal turnover in microbial community membership. At the phylum level, Pseudomonadota (formerly Proteobacteria) dominated the gut microbiota, but no significant difference was found between seasons (77.52% during hibernation vs. 57.15% during summer). Bacillota (formerly Firmicutes) decreased significantly, while Actinomycetota (formerly Actinobacteriota) increased significantly in the hibernation group compared to the summer group. Genus‐level composition exhibited seasonal variation, with distinct microbial communities characterizing each period. Alpha diversity analysis revealed significant differences in Faith's phylogenetic diversity between seasons, suggesting shifts in phylogenetic composition, while Chao1, Shannon, and Simpson indices remained unchanged. Beta diversity analyses revealed significant structural divergence between seasonal groups. Functional prediction using PICRUSt2 suggested seasonal shifts in metabolism‐related pathways, with putative enrichment of lipid metabolism and xenobiotic biodegradation pathways during hibernation, while carbohydrate metabolism appeared more prominent during the active period. These findings suggest that winter fasting may alter intestinal microbial metabolic functions, potentially shifting the microbiota from carbohydrate‐oriented to lipid‐oriented metabolism. This study enhances our understanding of host‐microbiome crosstalk in hibernating mammals and highlights the potential adaptive role of gut microbes in facilitating survival under extreme physiological conditions.

## Graphical abstract

The composition and dynamics of gut microbial community structure in *Rhinolophus sinicus* , employing 16S rRNA amplicon sequencing, are conducted to investigate the effects of physiological status (winter hibernation versus summer activity) on gut microbiota composition. Also, PICRUSt analysis was applied to predict functional characteristics of the gut microbiota under different seasonal conditions. These findings suggest that winter fasting alters intestinal microbial metabolic functions, potentially shifting the microbiota from carbohydrate‐oriented to lipid‐oriented metabolism.

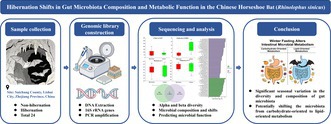

## 1 Introduction

The gut serves as a primary organ for nutrient absorption and immune function in animals (Wang, Sunday, et al. 2020). The gut microbiota comprises a vast and complex community of microorganisms inhabiting the digestive tract, including bacteria, viruses, fungi, and other microbial taxa (Brestoff and Artis 2013). In addition to priming and modulating the immune system in both humans and animals, the gut microbiota plays indispensable roles in digestion, absorption, and excretion to maintain physiological and biochemical homeostasis (Liu, Wang, and Wu 2022; Deng et al. 2025; He et al. 2025). The composition and function of gut microbial communities are influenced by numerous intrinsic and extrinsic factors, including diet, phylogeny, geography, age, and environmental conditions (Amato 2013; Li et al. 2016; Phillips et al. 2012). For instance, captive Guizhou snub‐nosed monkeys ( *Rhinopithecus brelichi* ) fed a relatively monotonous, low‐fiber diet exhibit reduced gut microbiota diversity due to limited nutritional substrates (Hale et al. 2019). In commercial fish species, gut microbiota diversity increases significantly during development and stabilizes following maturation and dietary stabilization (Li et al. 2017). Similarly, in plateau pikas ( *Ochotona curzoniae* ), both community abundance and coverage indices are significantly elevated during the non‐breeding season compared to the breeding season (Zhang et al. 2022). Furthermore, gut microbiota alterations have been associated with various diseases, including Alzheimer's disease and depression (Vogt et al. 2017; Wang, Sun, et al. 2020). Consequently, changes in gut microbiota composition may substantially affect host health status.

Hibernation represents one of the most extreme physiological challenges faced by heterothermic mammals (Regan et al. 2022). During hibernation, animals experience prolonged fasting, reduced metabolic rate, lowered body temperature, and suppressed immune function (Huus and Ley 2021; Carey et al. 2003). These dramatic physiological changes are likely to substantially alter the gut environment and, consequently, the gut microbial community (Regan et al. 2022; Grond et al. 2021). For instance, the microbiota of hibernating brown bear ( *Ursus arctos* ) is enriched with Bacteroidetes at the expense of Bacillota and Actinomycetota, which was experimentally shown to optimize energy preservation (Sommer et al. 2016). In Daurian ground squirrels ( *Spermophilus dauricus* ), microbial diversity was lower in the small intestine than in the caecum and large intestine during summer. Furthermore, the small intestine exhibited higher microbial diversity in winter compared to summer, while the caecum and large intestine showed the opposite trend (Zheng et al. 2025). In North American red squirrels ( *Tamiasciurus hudsonicus* ), limited dispersal could play a role in shaping and maintaining the structure of gut microbial communities. It also found a remarkable seasonal rhythm in the red squirrel's gut microbial composition manifested by a tradeoff between the relative abundance of two genera, *Oscillospira* and *Corpococcus*, and clearly associated with seasonal variation in diet availability (Ren et al. 2017). Similar patterns have been observed in 13‐lined ground squirrels ( *Ictidomys tridecemlineatus* ) that hibernation increased the relative abundance of *Bacteroidetes* and *Verrucomicrobia*, phyla that contain species capable of surviving on host‐derived substrates such as mucins, and reduced the relative abundance of Firmicutes, many of which prefer dietary polysaccharides (Carey et al. 2013), these above findings suggesting that hibernation‐associated gut microbiota restructuring may be a conserved phenomenon across diverse taxa.

Bats are nocturnal flying mammals capable of navigating and capturing prey in complete darkness (Yin et al. 2020; Bazzoni et al. 2024). Most bat species are insectivorous, with dietary composition varying considerably among species (Li et al. 2018; Dai et al. 2024). Due to their diverse feeding habits and broad ecological distribution, bats represent excellent model organisms for gut microbiota research (Yin et al. 2020; Dai et al. 2024). The composition and function of bat gut microbiota are influenced by multiple factors, including diet specializations (Phillips et al. 2012; Gong et al. 2021; Corduneanu et al. 2023; Mena Canata et al. 2024), gastrointestinal location (Zhou et al. 2016), age (Yin et al. 2020), season (Xiao et al. 2019), and time of day (Melville, Meyer, Kümmerle, et al. 2025). Additionally, bat microbiota may also play a role in tolerance or susceptibility to influenza and coronaviruses (Liu, Chen, et al. 2022; Melville, Meyer, Risely, et al. 2025). Interestingly, bat gut microbiota composition appears more similar to birds than to other mammals (Song et al. 2020), which raises the question as to whether bats exhibit similar gut microbiota responses to hibernation as other heterothermic mammals or alternative strategies yielding functionally similar outcomes.

The Chinese horseshoe bat ( *Rhinolophus sinicus* ) is a common insectivorous species in East Asia that undergoes seasonal hibernation. This cave‐dwelling species frequently roosts in large congregations (Mao et al. 2013; Ruedas 2006). Previous research on *R. sinicus* has focused on roosting ecology (Wu et al. 2022), echolocation calls, and genetic structure (Xie 2016), and SARS‐CoV virology (Guan et al. 2003). One published study examined gut microbiota demonstrating that the sampling source (large intestine, small intestine, and feces) influences alpha diversity of the microbial community in *R. sinicus* although no significant variations of beta diversity were observed (Wu et al. 2019). This finding may have been influenced by the small sample size (*n* = 3) and limited statistical power. The seasonal physiology variations in the composition and structure of the intestinal microbiota of this species remain unknown.

To address this knowledge gap, the present study employed 16S rRNA amplicon sequencing to investigate how physiological status (winter hibernation versus summer activity) affects gut microbiota composition in *R. sinicus* . We hypothesized that: (1) Hibernation would significantly alter gut microbial community composition and reduce diversity due to fasting conditions; (2) Hibernation would affect gut microbial taxonomic and predicted functional composition in metabolic functions, particularly from carbohydrate to lipid metabolism, reflecting the host's reliance on fat reserves during fasting. affects gut microbial taxonomic and predicted functional composition in.

## 2 Materials and Methods

### 2.1 Sample Collection

Twelve adult bats were collected during summer (non‐hibernation group, sampled on August 2, 2024) and winter (hibernation group, sampled on December 14, 2024) using mist nets and hand nets from Jinkuang Cave in Suichang County, Lishui City, Zhejiang Province, China (*n* = 12 per group, total *N* = 24). Each captured bat was individually placed in a clean cotton cloth bag. Bats were euthanized by cervical dislocation followed by immediate decapitation. Intact gastrointestinal tracts were rapidly excised within 10 min of euthanasia to minimize post‐mortem effects on microbial profiles. Intestinal contents were collected and immediately frozen in liquid nitrogen, then stored at −80°C until DNA extraction. All animal handling procedures complied with current Chinese laws on animal welfare and research. This study was approved by the Academic Committee of the College of Agriculture and Biotechnology, Lishui University (approval number: STXY‐AE‐201401).

### 2.2 DNA Extraction and PCR Amplification

Following the methods described by Xiao et al. (2019) and Liu et al. (2025), intestinal contents from each sample were homogenized, and 0.1 g of homogenized material was used for microbial genomic DNA extraction using the DNeasy PowerWater Kit (QIAGEN Inc., Netherlands) according to the manufacturer's protocol. DNA concentration was quantified using a NanoDrop spectrophotometer, and DNA quality was assessed by 0.8% agarose gel electrophoresis. The samples from summer and hibernation were divided into two batches for extraction.

The V3‐V4 hypervariable region of bacterial 16S rRNA genes was amplified using forward primer 338F (5′‐ACTCCTACGGGAGGCAGCA‐3′) and reverse primer 806R (5′‐GGACTACHVGGGTWTCTAAT‐3′) (Xiao et al. 2019; Zhang et al. 2022). Sample‐specific 7‐bp barcodes were incorporated into the primers to enable multiplex sequencing. Each 25 μL PCR reaction contained 10–20 ng genomic DNA, 1 μL each of forward and reverse primers, 0.25 μL NEB Q5 High‐Fidelity DNA Polymerase, 5 μL 5× Reaction Buffer, 5 μL 5× GC Buffer, 2 μL dNTP mix (10 mM), and 8.75 μL sterile H_2_O. Negative PCR controls were included in each batch. Thermal cycling conditions consisted of initial denaturation at 95°C for 3 min, followed by 35 cycles of 94°C for 30 s, 55°C for 30 s, and 72°C for 30 s, with a final extension at 72°C for 10 min. Following Gong et al. (2021), PCR products were visualized by electrophoresis on 2.0% agarose gels. Amplicons were purified using Agencourt AMPure XP beads (Beckman Coulter, Indianapolis, IN, USA) and quantified with the Qubit 3.0 DNA Detection Kit (Life Technologies, Carlsbad, CA, USA). Purified amplicons were pooled in equimolar ratios and subjected to paired‐end sequencing (2 × 300 bp) on the Illumina NextSeqTM 2000 platform according to standard protocols at Wuhan Tianyi Huayu Gene Technology Co. Ltd. (Wuhan, China).

### 2.3 Sequence Data Processing

Microbial raw sequences were merged by FLASH (version 1.2.7; http://www.cbcb.umd.edu/software/flash) and processed using QIIME2 (version 2019.4; https://docs.qiime2.org/2019.4/tutorials/). We used the DADA2 plugin in QIIME2 to remove low‐quality reads (including non‐amplified regions, non‐bacterial sequences from chloroplasts and mitochondria), low‐complexity reads, and adapter‐contaminated sequences. These steps filtered out noise, removed chimeras and singletons, and finally resulted in a series of high‐resolution ASVs and a feature table of ASV counts for subsequent analysis (Gong et al. 2021). Taxonomic assignment was performed to the ASV feature table using the Native Bayes classifier in QIIME2 trained against the SILVA reference database, version 138 (https://www.arb‐silva.de/no_cache/download/archive/release_138/Exports/) (Zhang et al. 2022; Li et al. 2025). To remove the influences of variable sequencing depth, we rarefied the ASV feature table to 20,000 sequences per sample according to the produced rarefaction curves in QIIME2 for downstream analyses (Gong et al. 2021).

### 2.4 Data Analysis

#### 2.4.1 Alpha and Beta Diversity Analyses

Alpha diversity indices, including Shannon, Chao1, Simpson, and Faith's phylogenetic diversity were calculated using QIIME software. ASV‐level ranked abundance curves and rarefaction curves were generated to assess sampling adequacy (Yang et al. 2024). Differences in alpha diversity metrics between summer and winter groups were compared using Mann–Whitney *U* tests in R version 4.2.0 (Yin et al. 2020).

To analyze the structure of the gut microbial community between seasons, we performed beta diversity analysis. Principal coordinate analysis (PCoA) plots were generated using three distance metrics: Bray‐Curtis, unweighted UniFrac, and weighted UniFrac distances (Lozupone and Knight 2005; Lozupone et al. 2007; Oksanen et al. 2019). Statistical significance of beta diversity differences was assessed using permutational multivariate analysis of variance (PERMANOVA) with 999 permutations implemented in the vegan package in R version 3.5.0 (Oksanen et al. 2019). Additionally, permutational analysis of multivariate dispersions (PERMDISP) with 999 permutations was performed to test homogeneity of dispersions using the betadisper function in the vegan package R version 3.5.0 (Anderson and Walsh 2013).

#### 2.4.2 Microbial Composition and Shifts Analyses

Based on the results of the taxonomic analysis, changes in relative abundances of gut microbial taxa between seasons at the phylum and genus levels were visualized using QIIME2. Updated bacterial nomenclature following the Genome Taxonomy Database (GTDB) was used throughout (e.g., Pseudomonadota for Proteobacteria, Bacillota for Firmicutes, Actinomycetota for Actinobacteriota) (Risely 2020). Venn diagrams were generated to display shared ASVs between the two seasonal groups. Linear discriminant analysis effect size (LEfSe) was used to identify differentially abundant bacterial taxa between groups, with a linear discriminant analysis (LDA) score > 2 indicating significant differences (Gong et al. 2021; Segata et al. 2011).

#### 2.4.3 Predicting Changes in Microbial Function via PICRUSt2

To compare changes in microbial function associated with seasonal shifts, functional metagenomic prediction analysis by means of PICRUSt2 was performed on the ASVs within the QIIME2 environment (Douglas et al. 2020). Predicted metagenome data were obtained using a rarefied ASV feature table (20,000 sequences per sample). *t*‐tests were used to compare the relative abundance of metabolic functional categories using the second‐level KEGG pathways between hibernation and summer active groups.

## 3 Results

### 3.1 Sequencing Data Overview

**FIGURE 1:**
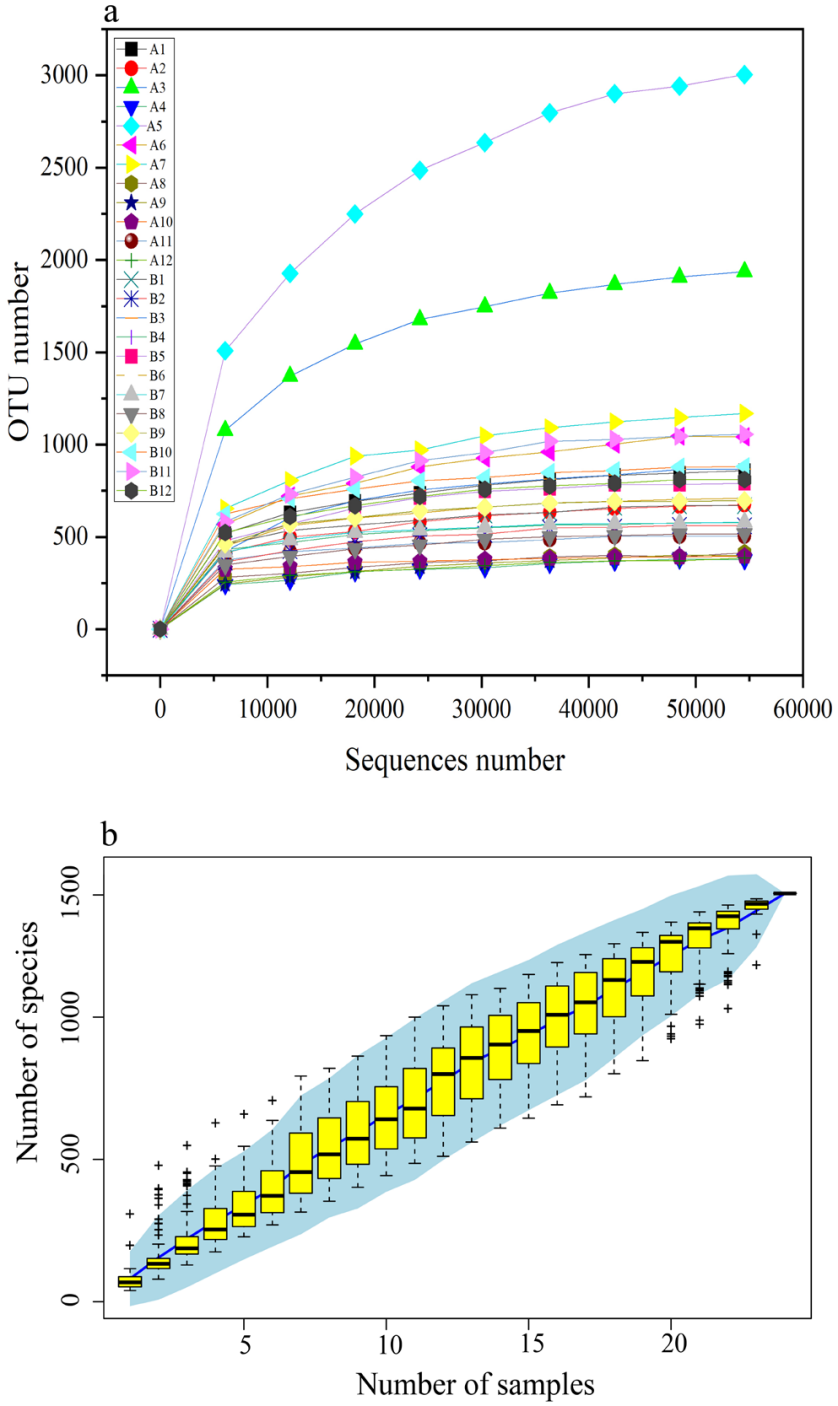
Assessment of sampling adequacy for gut microbiota analysis. (a) Rarefaction curves (b) Species accumulation curves.

Following quality control, sequencing yielded a total of 2,129,820 high‐quality reads, with an average of 88,743 sequences per sample (range: 63,193–114,810) (Table S1). Both rarefaction curves (Figure 1a) and species accumulation curves (Figure 1b) approached asymptotes, indicating that sampling depth was sufficient to capture the majority of microbial diversity present in the intestinal samples, although some rare taxa may remain undetected. A total of 1,858,124 high‐quality, non‐chimeric sequences were classified into 1435 ASVs spanning 22 bacterial phyla across all 24 samples.

### 3.2 Alpha and Beta Diversity

**FIGURE 2:**
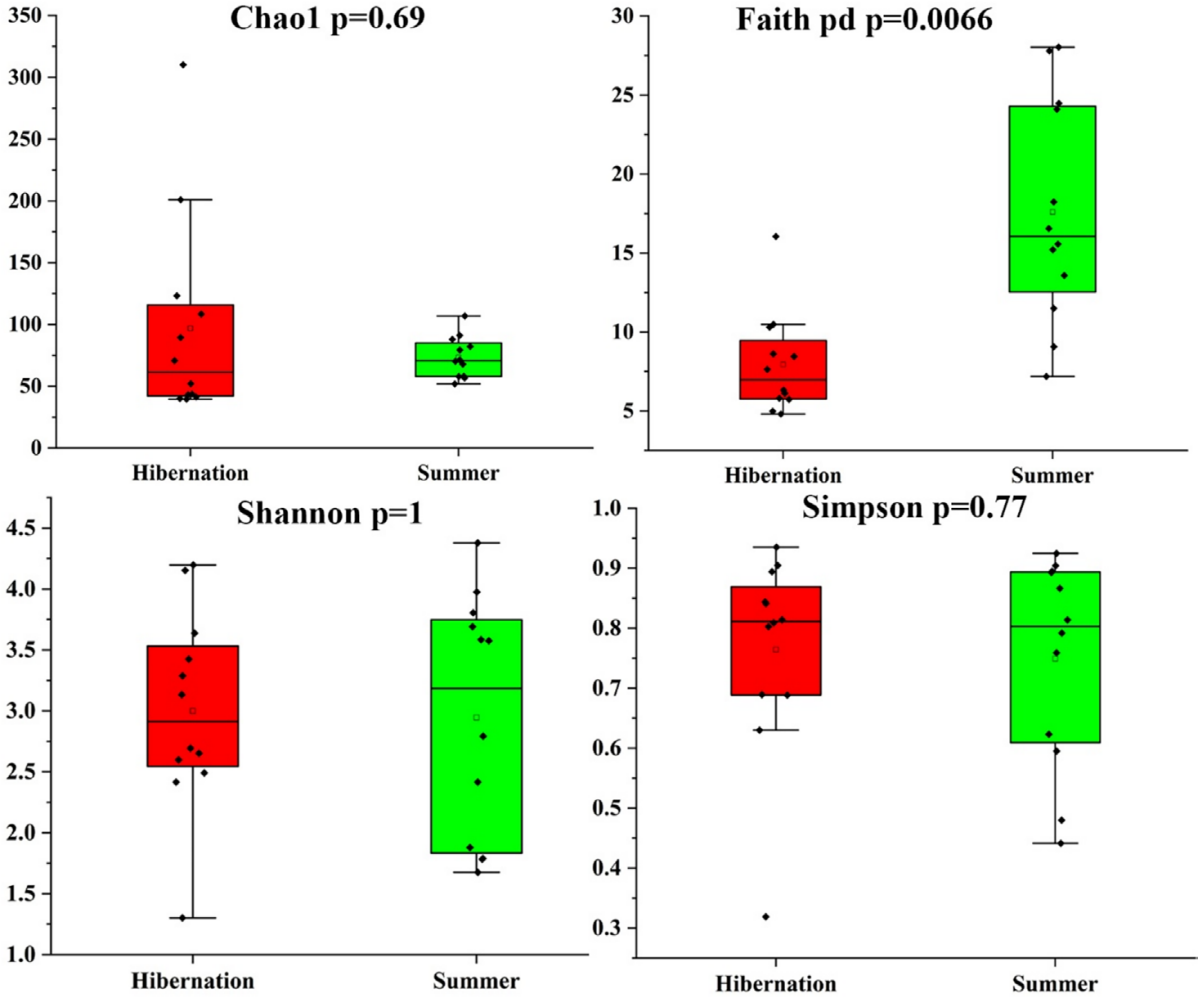
Alpha diversity metrics of gut microbiota in *R. sinicus* . Box plots comparing four alpha diversity indices between hibernation and summer active groups.

Alpha diversity metrics are presented in Figure 2. Significant differences between hibernation and summer groups were observed only for Faith's phylogenetic diversity (*p* = 0.00066). The significant difference in Faith's phylogenetic diversity suggests a substantial shift in phylogenetic composition between seasons, while the lack of significant differences in Shannon diversity indices indicates that relative abundance distributions remained similar. The remaining two alpha diversity metrics showed no significant differences between the two seasonal groups.

**FIGURE 3:**
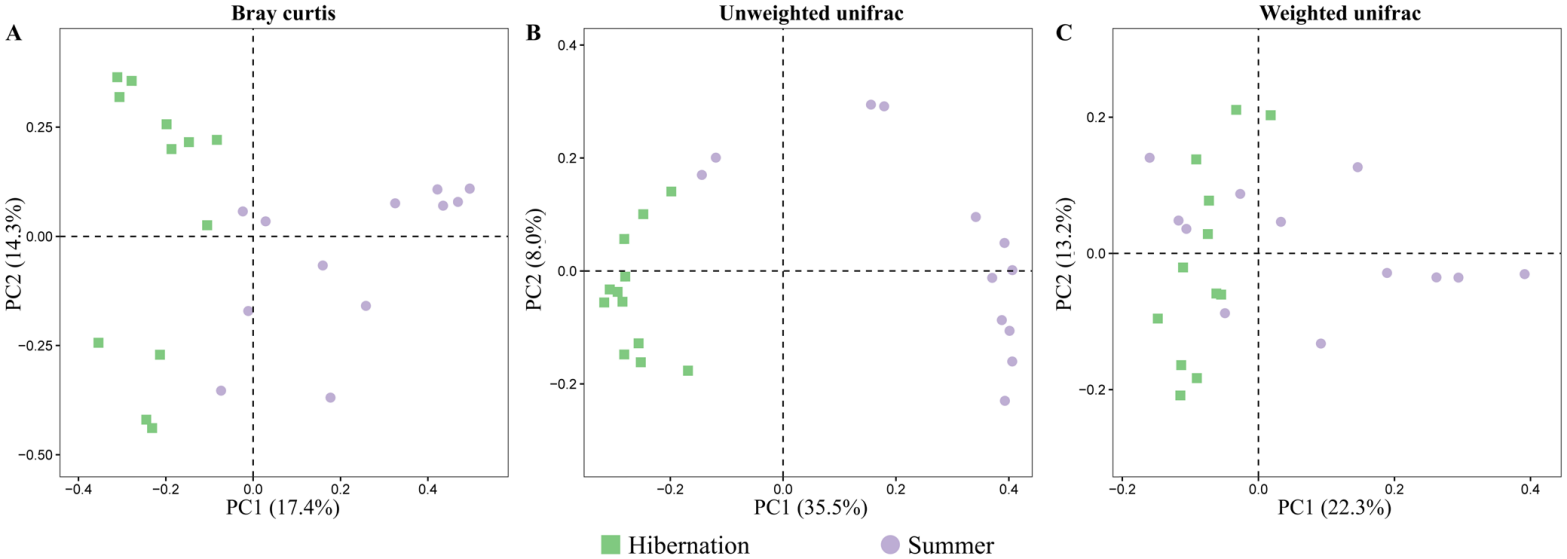
Beta diversity analysis of gut microbial communities. Principal coordinate analysis (PCoA) plots based on (A) Bray‐Curtis distance, (B) unweighted UniFrac distance, and (C) weighted UniFrac distance.

Beta diversity analysis revealed significant differences in gut microbial community structure between hibernation and summer groups (Figure 3). Principal coordinate analysis (PCoA) based on Bray‐Curtis distance (PERMANOVA: *R* ^2^ = 0.1498, *p* = 0.001; Figure 3A), unweighted UniFrac distance (PERMANOVA: *R* ^2^ = 0.2876, *p* = 0.001; Figure 3B), and weighted UniFrac distance (PERMANOVA: *R* ^2^ = 0.1473, *p* = 0.014; Figure 3C) clearly demonstrated seasonal clustering of gut microbial communities. Furthermore, PERMDISP analysis showed that Bray‐Curtis distance (*F* = 2.551, *p* = 0.062; Figure S1A), unweighted UniFrac distance (*F* = 0.027, *p* = 0.805; Figure S1B), and weighted UniFrac distance (*F* = 1.556, *p* = 0.228; Figure S1C) were homogeneous dispersion.

**FIGURE 4:**
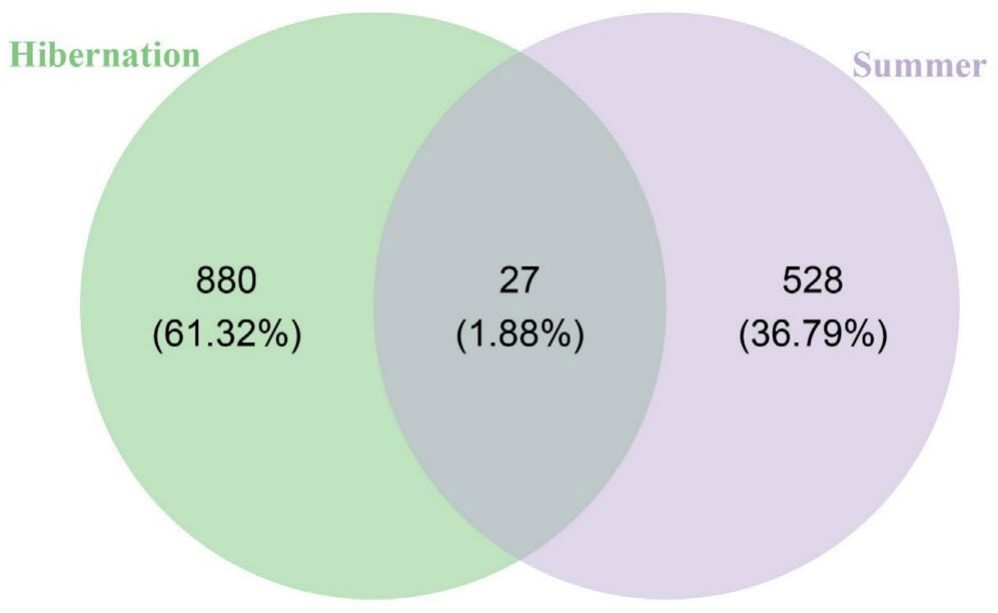
Venn diagram illustrating shared and unique ASVs between seasonal groups.

Moreover, only 27 ASVs were shared between the 907 ASVs found in the hibernation group and the 555 ASVs identified in the summer group, underscoring a substantial seasonal turnover in microbial community membership (Figure 4).

### 3.3 Gut Microbiota Composition and Seasonal Variation

**FIGURE 5:**
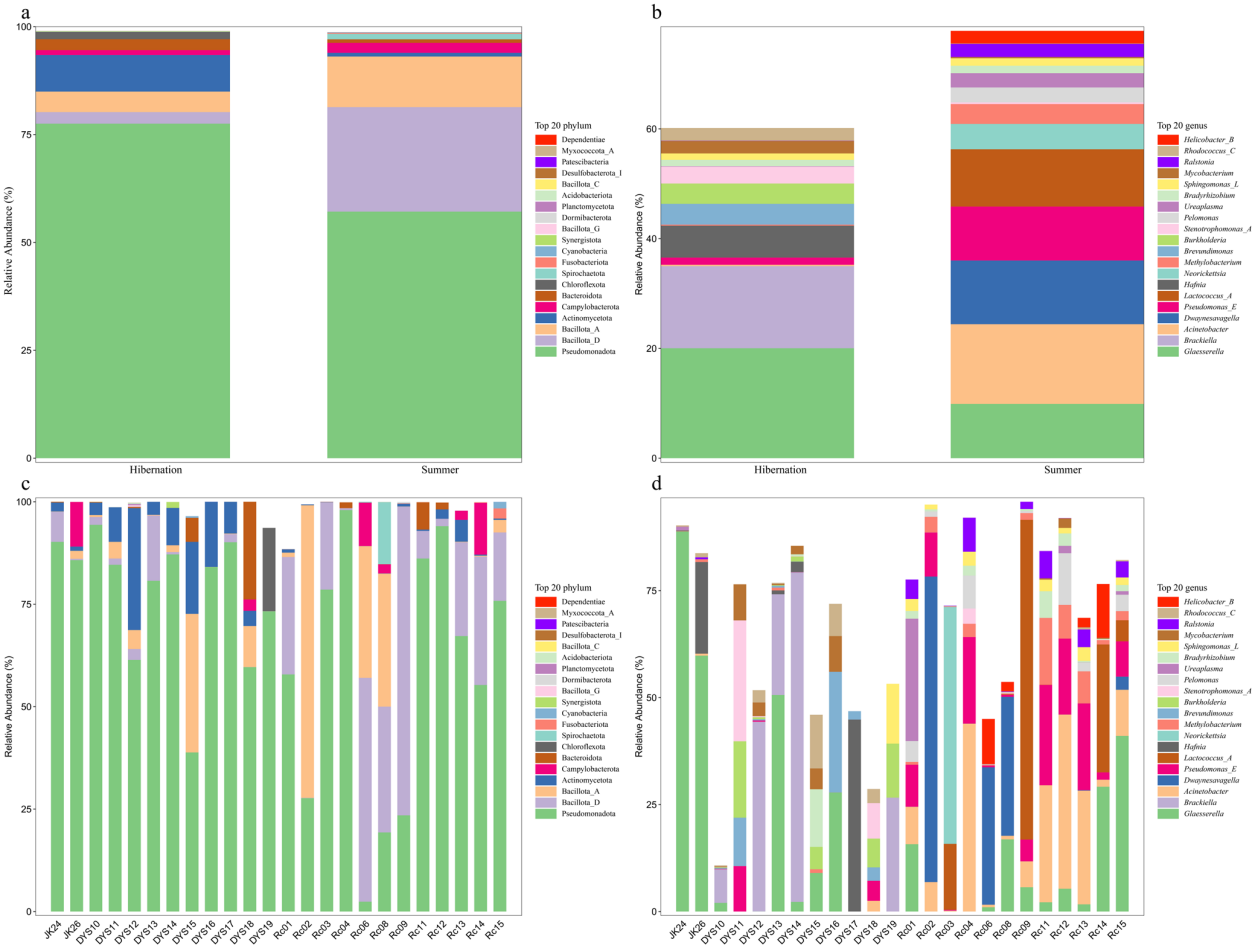
Taxonomic composition of gut microbiota in *R. sinicus* . Relative abundance of bacterial taxa at the phylum level showing (a) average composition by group and (c) individual variation across all bats. Genus‐level composition showing (b) average composition by group and (d) individual variation across all bats. Only taxa with relative abundance > 1% are displayed.

Taxonomic composition analysis is presented in Figure 5. Results revealed that Pseudomonadota (formerly Proteobacteria) was the dominant phylum, constituting a core microbiome component across all individual bats (Figure 5a,c). At the phylum level, Pseudomonadota exhibited relative abundances of 77.52% in the hibernation group and 57.15% in the summer group, followed by Bacillota (formerly Firmicutes; 7.44% vs. 35.92%), Actinomycetota (formerly Actinobacteriota; 8.47% vs. 0.86%), and Campylobacterota (1.14% vs. 2.30%) in the respective groups.

At the genus level, distinct compositional differences in gut microbial communities were observed between seasons (Figure 5b,d). In the hibernation group, the dominant genera were *Glaesserella* (20.03%), *Brackiella* (14.95%), unclassified Enterobacteriaceae (10.69%), and *Hafnia* (5.82%). In contrast, the summer group was dominated by *Acinetobacter* (14.51%), *Dwaynesavagella* (11.61%), *Lactococcus* (10.44%), *Glaesserella* (9.90%), unclassified Metamycoplasmataceae (6.99%), and *Neorickettsia* (4.62%).

**FIGURE 6:**
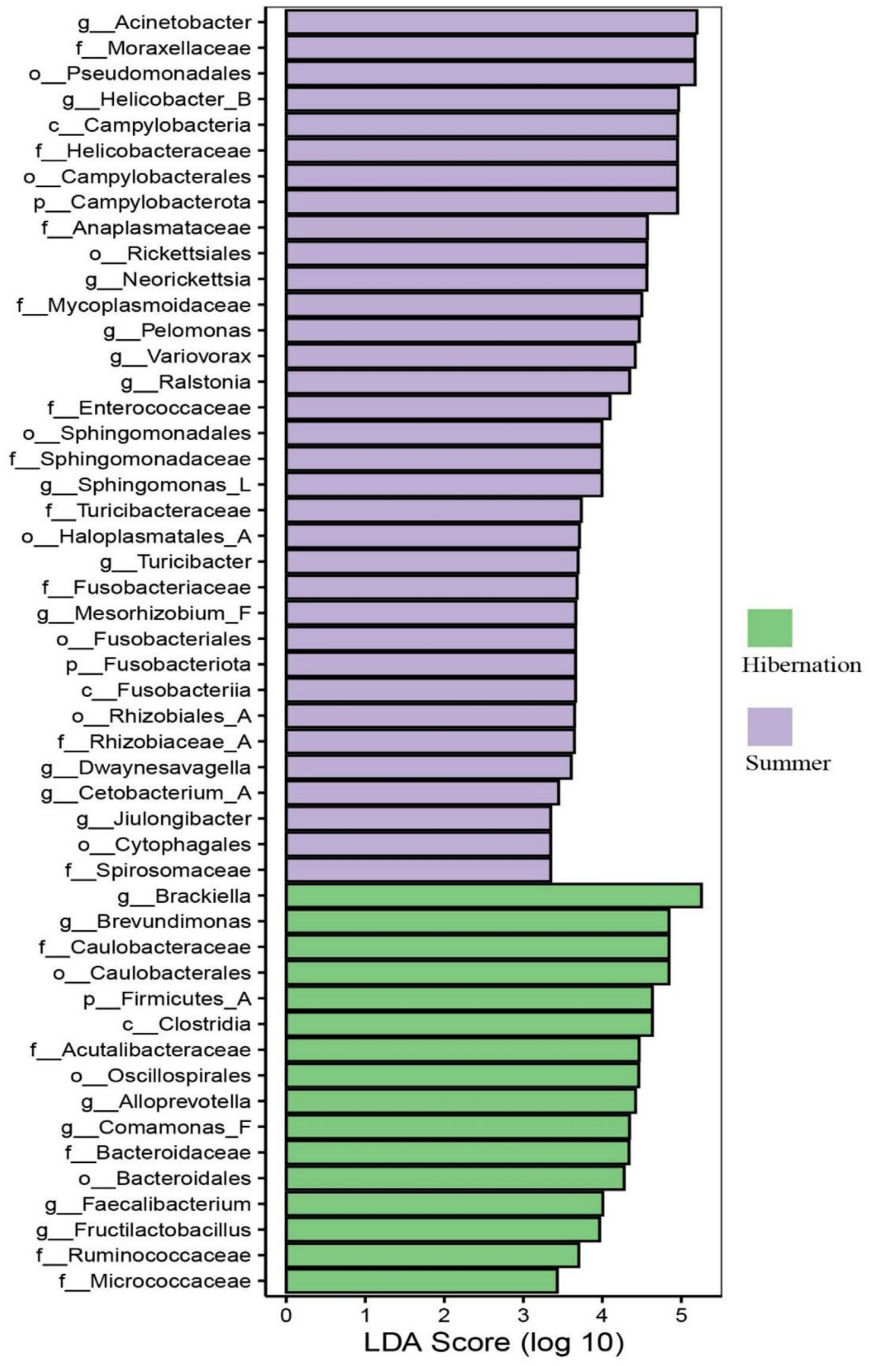
Cladogram from Linear Discriminant Analysis Effect Size (LEfSe) showing taxa with significantly different abundances at multiple taxonomic levels (LDA score > 3, *p* < 0.05). Taxa enriched in hibernation group are shown in green; those enriched in summer group are shown in pale pink.

Linear discriminant analysis effect size (LEfSe) revealed taxa with significantly different relative abundances between groups at multiple taxonomic levels (LDA score > 3, *p* < 0.05; Figure 6). At the phylum level, three phyla showed significant differences: Bacillota was enriched in the hibernation group, while Campylobacterota and Fusobacteriota were enriched in the summer group. At the genus level, 18 genera exhibited significant differences. Genera enriched in the hibernation group included *Brackiella*, *Brevundimonas*, *Alloprevotella*, *Comamonas*, *Faecalibacterium*, and *Fructilactobacillus*, whereas those enriched in the summer group included *Acinetobacter*, *Helicobacter*, *Neorickettsia*, *Pelomonas*, *Variovorax*, *Ralstonia*, *Sphingomonas*, *Turicibacter*, *Mesorhizobium*, *Dwaynesavagella*, *Cetobacterium*, and *Jiulongibacter*.

### 3.4 Functional Prediction Profiles of Gut Microbiota

Functional prediction analysis using PICRUSt2 revealed similar relative abundances of functional genes in the intestinal microbiota between hibernation and summer groups. First‐level KEGG pathway analysis identified six core functional domains: metabolism, genetic information processing, environmental information processing, cellular processes, human diseases, and organismal systems. Notably, approximately 80% of functional genes were enriched in metabolic pathways (Figure S2), suggesting the central role of the gut microbial community in digestive metabolism in *R. sinicus* .

**FIGURE 7:**
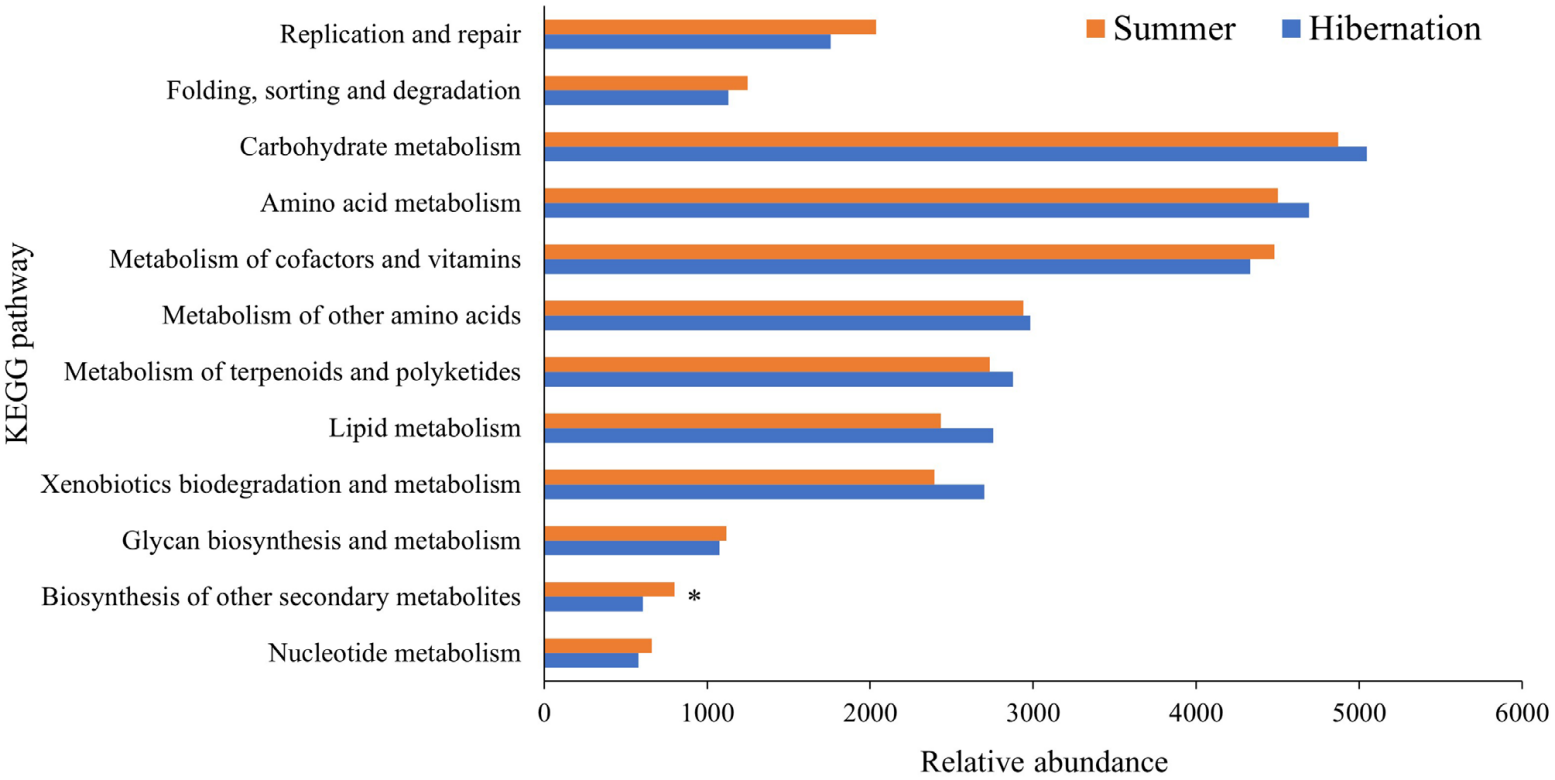
Functional predictions of gut microbiota at KEGG level 2 pathways. *p < 0.05.

Second‐level pathway analysis showed that predicted genes were predominantly enriched in carbohydrate metabolism, amino acid metabolism, and metabolism of cofactors and vitamins within the metabolism category, while replication and repair, as well as folding, sorting, and degradation pathways were mainly enriched within the genetic information processing category (Figure 7). Although predicted functional gene profiles were generally similar between seasonal groups, notable differences were observed: xenobiotic biodegradation and metabolism, and lipid metabolism pathways were higher in the hibernation group, while biosynthesis of other secondary metabolites increased significantly in the summer group (Figure 7).

## 4 Discussion

### 4.1 Sequencing Depth and Data Quality

The present study obtained an average of 88,743 high‐quality sequences per sample, substantially exceeding the sequencing depth reported in previous bat gut microbiota studies, including *Vespertilio sinensis* (51,733 sequences; Yin et al. 2020), *Rhinolophus ferrumequinum* (34,041 sequences; Xiao et al. 2019), *Hipposideros pratti* (29,355 sequences; Jia 2022), and *H. caffer* (32,849 sequences; Melville, Meyer, Risely, et al. 2025). This enhanced sequencing depth provides a solid foundation for robust characterization of the gut microbial communities. However, the considerable inter‐individual variability observed in our data, as reflected in the low number of shared ASVs (27 out of 1435 total) and the wide range of variable beta diversity distances among individuals. This variability suggests that future studies would benefit from larger sample sizes to fully capture population‐level patterns.

### 4.2 Core Gut Microbiota Composition

The composition of the intestinal microbiota of animals changes significantly with the seasons, which is mainly attributed to the adjustment of seasonal food resource structure affecting the intestinal microbiota (Ur Rahman Shah et al. 2025). Our analysis of seasonal variation in *R. sinicus* gut microbiota revealed that Pseudomonadota was the dominant phylum, consistent with typical bat intestinal microbiota characteristics. This dominance of Pseudomonadota aligns with findings from other bat species, including *Mops condylurus* (Edenborough et al. 2020), *R. ferrumequinum* (Xiao et al. 2019), and *V. sinensis* (Yin et al. 2020), demonstrating the evolutionary conservation of bat gut microbiota. Notably, bat gut microbiota appears more similar to birds than to other mammals in its Pseudomonadota‐dominated composition (Song et al. 2020), raising interesting questions about whether bats exhibit convergent gut microbiota responses to hibernation with other mammals despite these compositional differences.

Compared with other mammals, adaptive adjustments of the bat intestinal microbiota may play a pivotal role in the adaptive evolution of bat populations, thereby providing support for maintaining physiological homeostasis in a complex environment (Bazzoni et al. 2024). Many bat species enter a state of hibernation during cold seasons to cope with the low temperatures and food scarcity, and changes in the intestinal microbiota may play an important role in the energy metabolism and immune regulation of bats during hibernation (Geluso 2007). For example, a study of *Nyctalus plancyi* found that its intestinal microbiota α‐diversity significantly decreased during hibernation and gradually recovered to pre‐hibernation levels after waking up in early spring (Popov et al. 2023). Another study that sequenced the 16S rRNA gene of the intestinal microbiota of *R. ferrumequinum* has shown that seasonal changes in food significantly affect the relative abundance of dominant phyla, specifically the abundance of Pseudomonadota (formerly Proteobacteria) being highest during early summer; while the Bacillota (formerly Firmicutes) phylum was least abundant during early summer, increased toward the end of summer, and then significantly reduced during early winter and early spring, reflecting the regulatory changes in food resources on the dominant phyla (Xiao et al. 2019). Another study also found that differences in environmental temperature during hibernation can significantly alter the α‐diversity of the intestinal microbiota and related energy metabolism pathways, but do not alter the β‐diversity of the intestinal microbiota, which also highlights the importance of intestinal microbiota stability for the bat hibernation process (Liu et al. 2023).

In contrast, other small hibernating mammals such as black‐tailed prairie dogs ( *Cynomys ludovicianus* ) (Neha and Salazar‐Bravo 2023), Daurian ground squirrels ( *Spermophilus dauricus* ) (Song et al. 2023), thirteen‐lined ground squirrels ( *Ictidomys tridecemlineatus* ) (Carey et al. 2013), and brown bears (Sommer et al. 2016) are dominated by Bacillota and Bacteroidota, with Pseudomonadota representing only a minor component, highlighting that seasonal changes in the intestinal microbiome may regulate the metabolic patterns of hibernating animals. These findings indicate that the dominant gut microbiota composition differs fundamentally between bats and other hibernating mammals, making comparative analyses of hibernation responses particularly valuable.

### 4.3 Seasonal Shifts in Gut Microbiota Composition

Comparison of gut microbial composition between seasons revealed significant shifts in the relative abundance of major bacterial phyla. Pseudomonadota increased from 57.15% in summer to 77.52% in winter, while Bacillota decreased significantly from 35.92% to 7.44%, and Actinomycetota increased markedly from 0.86% to 8.47%. Similar hibernation‐associated changes have been documented in other species. In *R. ferrumequinum* , Bacillota decreased while Tenericutes increased during hibernation (Xiao et al. 2019). In ground squirrels, Bacillota decreased while Bacteroidota and Verrucomicrobia increased (Carey et al. 2013). In brown bears, hibernation was associated with reductions in microbial diversity and shifts in community composition (Sommer et al. 2016).

Although the dominant phyla differ between *R. sinicus* and other hibernating mammals, convergent patterns emerge in gut microbiota responses to hibernation. The decrease in Bacillota observed in our study parallels changes in other hibernators, suggesting common adaptive mechanisms despite different baseline compositions. The relationship between hibernation and gut microbiota is also interconnected with host immunity, as seasonal challenges including hibernation have been shown to influence both immune function and gut microbial communities (Schmid et al. 2023). These findings suggest that gut microbiome alterations in *R. sinicus* and other hibernating animals may represent similar adaptive responses to hibernation (Xiao et al. 2019), though the specific microbial players involved differ among taxa. Convergent patterns may arise from similar ecological pressures (prolonged fasting, reduced body temperature) even when the starting microbial communities differ substantially.

### 4.4 Factors Influencing Gut Microbiota Diversity

Multiple factors likely contribute to the seasonal differences in gut microbiota observed in *R. sinicus* . During hibernation, bats rely primarily on stored body fat to minimize energy expenditure (Yu et al. 2014), with body temperature dropping to slightly above ambient roosting temperature. *R. sinicus* frequently congregates in large groups during hibernation, with individuals clinging together and wrapping wing membranes tightly around their bodies in deep torpor, further reducing energy consumption (Wu et al. 2022). These hibernation‐associated physiological changes, including fasting, reduced body temperature, and altered gut motility, substantially alter the intestinal environment, potentially impacting gut microbial communities (Xiao et al. 2019). However, the relative contributions of dietary cessation, temperature reduction, and other physiological changes to the observed microbiota differences remain to be determined through controlled experiments.

### 4.5 Putative Functional Implications of Seasonal Microbiota Changes

PICRUSt2 functional prediction suggested similar overall microbiota functions between seasonal groups, with approximately 80% of predicted genes associated with metabolic pathways. However, some differences were observed: predicted lipid metabolism and xenobiotic biodegradation pathways were higher in the hibernation group, while biosynthesis of secondary metabolites appeared higher during the active period. These putative functional differences may reflect a shift from dietary carbohydrate utilization during the active season to endogenous lipid catabolism during fasting hibernation, consistent with patterns reported in other hibernating mammals (Xiao et al. 2019; Carey et al. 2013). However, these findings are based solely on predicted metagenomes inferred from 16S rRNA marker genes, and validation through shotgun metagenomics or metabolomics would be required to confirm actual functional differences.

### 4.6 Study Limitations

Several limitations of this study should be acknowledged. First, the sample size (*n* = 12 per group) is relatively small for microbiome studies, which may limit statistical power and the ability to detect subtle community differences. The high inter‐individual variability observed, as reflected in the low number of shared ASVs and modest *R* ^2^ values in PERMANOVA, underscores the need for larger sample sizes in future studies. Second, this study represents a cross‐sectional comparison between two seasonal groups rather than longitudinal tracking of the same individuals, so we cannot definitively attribute observed differences to hibernation versus individual variation. The use of terms such as “increase” or “decrease” should be interpreted as differences between groups rather than changes within individuals. Third, our functional analyses rely on PICRUSt2 predictions based on 16S rRNA marker genes, which provide only inferred rather than directly measured functional profiles. The accuracy of these predictions depends on the representation of bat‐associated bacteria in reference databases, which may be limited. Future studies employing shotgun metagenomics or metabolomics would provide more definitive functional insights. Fourth, the 16S rRNA V3‐V4 region has inherent taxonomic resolution limitations, particularly at the species level, as reflected in the high proportion of unclassified genera in our data. Finally, because summer and hibernation samples were extracted in separate batches, batch effects cannot be entirely ruled out as a contributing factor to the observed differences. Although negative PCR controls were included in each batch and no amplification was observed, a formal decontamination analysis (e.g., using the decontam package) was not performed. Consequently, we cannot completely exclude the possibility that some observed differences reflect batch‐related technical variation rather than true biological differences. Future studies should ideally process all samples within a single extraction batch or employ statistical methods to assess and correct for potential batch effects.

## 5 Conclusion

This study demonstrated significant seasonal variation in the diversity and composition of gut microbiota in *R. sinicus* between summer activity and winter hibernation periods. Pseudomonadota dominated the gut microbiota in both conditions, while Bacillota was substantially lower and Actinomycetota was higher during hibernation compared to summer. Faith's phylogenetic diversity differed significantly between seasons, suggesting shifts in phylogenetic composition, while evenness indices remained unchanged. These patterns are partially consistent with hibernation responses observed in other mammals, despite the different baseline microbiota composition in bats compared to typical mammalian hibernators.

Functional prediction using PICRUSt2 suggested seasonal shifts in metabolism‐related pathways, with putatively increased lipid metabolism during hibernation, potentially reflecting the host's reliance on fat reserves during fasting. However, these predicted functional changes require validation through direct metagenomic or metabolomic measurements.

From a conservation perspective, understanding how gut microbiota respond to seasonal physiological challenges may provide insights into bat health and resilience, particularly as climate change alters seasonal timing and hibernation patterns. Future studies with larger sample sizes, longitudinal sampling designs, and complementary metagenomic and metabolomic approaches will be essential to fully characterize the functional significance of hibernation‐associated gut microbiota shifts and their implications for host physiology and conservation.

## Author Contributions

**Weiwei Shao:** investigation (equal), writing – original draft (equal). **Yalei Li:** investigation (equal), writing – review and editing (equal). **Xiaoyun Cheng:** investigation (equal), resources (equal), visualization (equal). **Ling Guo:** investigation (equal), methodology (equal), software (equal). **Li Wei:** conceptualization (equal), supervision (equal).

## Funding

This study was supported by the Key Research Projects of Lishui City (2021ZDYF05).

## Conflicts of Interest

The authors declare no conflicts of interest.

## Supporting information


**Table S1:** Sequencing data collected from 24 individuals.
**Figure S1:** Principal‐coordinate analysis (PCoA) plots of permutational analysis of multivariate dispersions (PERMDISP).
**Figure S2:** Functional profiles of gut microbiota at KEGG level 1 pathways.

## Data Availability

Raw sequence data have been submitted to the National Center for Biotechnology Information (NCBI) Sequence Read Archive under accession number PRJNA1393037.
