# Supplementary material for: Hibernation Shifts in Gut Microbiota Composition and Metabolic Function in the Chinese Horseshoe Bat (Rhinolophus sinicus)
Source: Ecol Evol. 2026 Feb 10;16(2):e73087. doi: 10.1002/ece3.73087 (PMC12891815; doi:10.1002/ece3.73087)
Supplement: Supplementary file 1 — Table S1: Sequencing data collected from 24 individuals. Figure S1: Principal‐coordinate analysis (PCoA) plots of permutational analysis of multivariate dispersions (PERMDISP). Figure S2: Functional profiles of gut microbiota at KEGG level 1 pathways. [file ECE3-16-e73087-s001.docx]

Table S1 Sequencing data collected from 24 individuals

| SampleID | Input | Filtered | Denoised | Merged | Non-chimeric | Non-singleton | Group |
| --- | --- | --- | --- | --- | --- | --- | --- |
| JK24 | 100682 | 95348 | 95031 | 94413 | 90878 | 90871 | A |
| JK26 | 99291 | 94919 | 94611 | 93498 | 79895 | 79893 | A |
| DYS10 | 102868 | 98226 | 97731 | 95874 | 76789 | 76763 | A |
| DYS11 | 102070 | 97440 | 97112 | 96429 | 92590 | 92585 | A |
| DYS12 | 107615 | 101360 | 100321 | 95367 | 75977 | 75932 | A |
| DYS13 | 100397 | 95053 | 94701 | 93170 | 80732 | 80726 | A |
| DYS14 | 109088 | 103474 | 103045 | 101509 | 91397 | 91389 | A |
| DYS15 | 107365 | 97975 | 97683 | 97267 | 96313 | 96312 | A |
| DYS16 | 114810 | 108873 | 108628 | 108148 | 105562 | 105559 | A |
| DYS17 | 98726 | 93948 | 93649 | 93217 | 90029 | 90028 | A |
| DYS18 | 105917 | 101014 | 100721 | 100069 | 99114 | 99112 | A |
| DYS19 | 107820 | 102768 | 102593 | 102157 | 99804 | 99804 | A |
| Rc01 | 80859 | 76736 | 76368 | 75356 | 73316 | 73313 | B |
| Rc02 | 79688 | 75954 | 75726 | 73736 | 71701 | 71698 | B |
| Rc03 | 75926 | 72469 | 72148 | 71622 | 63790 | 63785 | B |
| Rc04 | 68549 | 64848 | 64666 | 64319 | 63898 | 63898 | B |
| Rc06 | 67460 | 63421 | 63156 | 62649 | 57900 | 57895 | B |
| Rc08 | 66159 | 63231 | 62993 | 62168 | 57414 | 57411 | B |
| Rc09 | 63815 | 60687 | 60430 | 59964 | 59009 | 59006 | B |
| Rc11 | 63193 | 59195 | 59049 | 58647 | 58153 | 58153 | B |
| Rc12 | 70395 | 66246 | 66005 | 65284 | 64924 | 64921 | B |
| Rc13 | 86800 | 82523 | 82199 | 81336 | 79592 | 79586 | B |
| Rc14 | 74948 | 71583 | 71154 | 70071 | 62256 | 62251 | B |
| Rc15 | 75379 | 71419 | 71074 | 70051 | 67091 | 67088 | B |


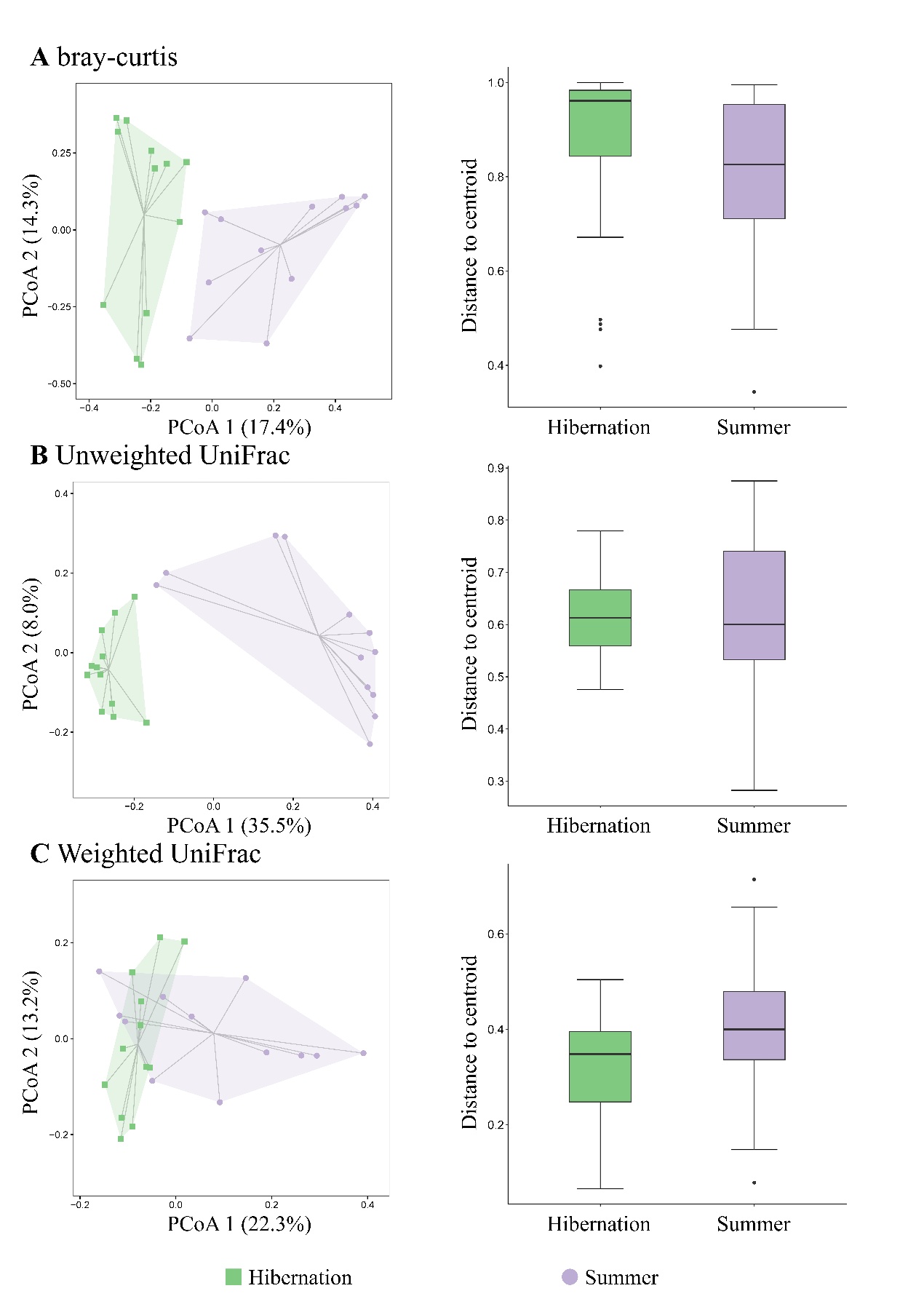


Figure S1 Principal-coordinate analysis (PCoA) plots of permutational analysis of multivariate dispersions (PERMDISP): Bray-Curtis distance (A), unweighted UniFrac distance (B), and weighted UniFrac distance(C).


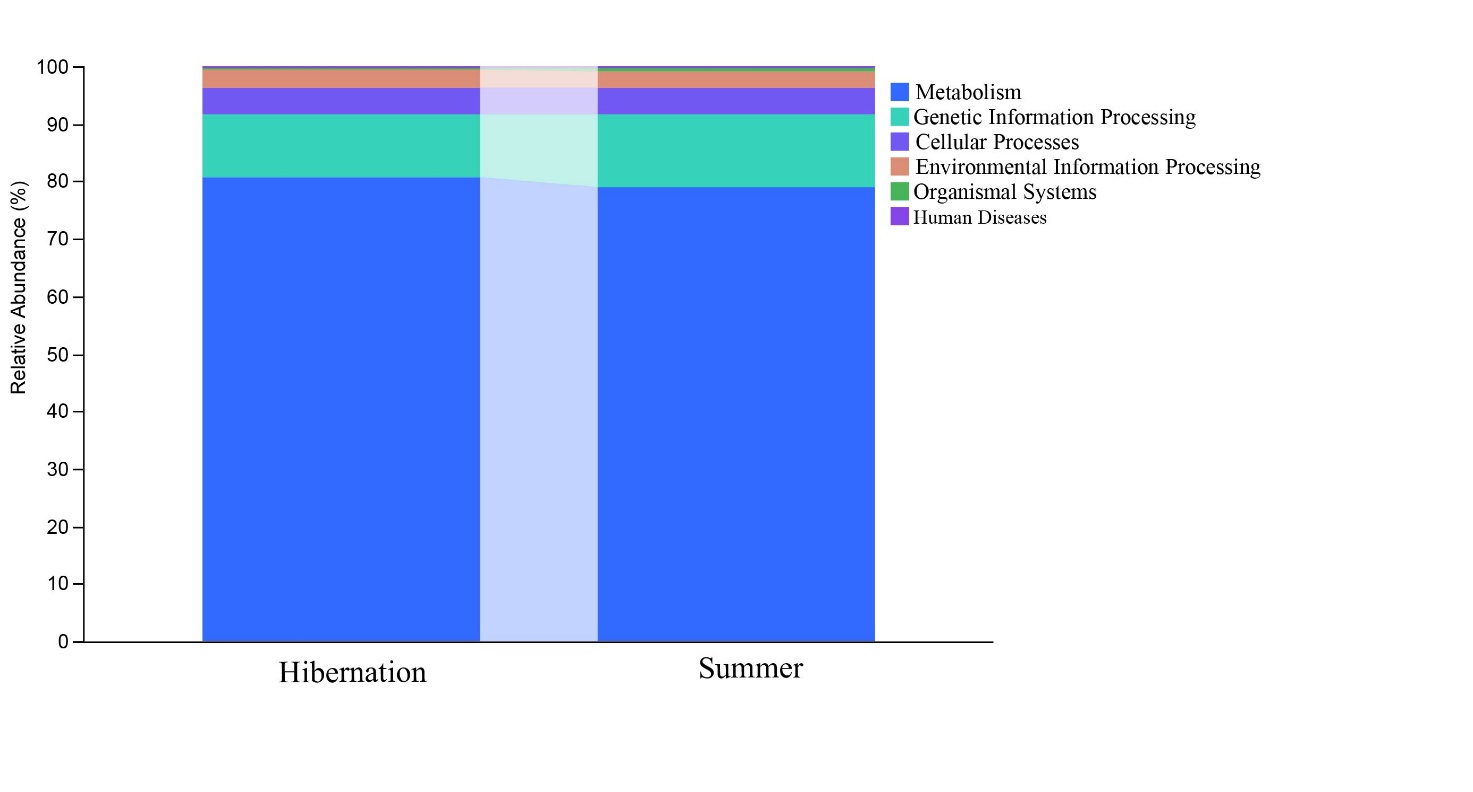


Figure S2 Functional profiles of gut microbiota at KEGG level 1 pathways.
